# Actionable Potentials of Less Frequently Mutated Genes in Colorectal Cancer and Their Roles in Precision Medicine

**DOI:** 10.3390/biom10030476

**Published:** 2020-03-20

**Authors:** Ryia Illani Mohd Yunos, Nurul Syakima Ab Mutalib, Francis Yew Fu Tieng, Nadiah Abu, Rahman Jamal

**Affiliations:** UKM Medical Molecular Biology Institute (UMBI), Universiti Kebangsaan Malaysia, Cheras, Kuala Lumpur 56000, Malaysia; ryia.yunos@ppukm.ukm.edu.my (R.I.M.Y.); francistieng@yahoo.com.my (F.Y.F.T.); nadiah.abu@ppukm.ukm.edu.my (N.A.)

**Keywords:** colorectal cancer, less frequently mutated genes, chemoresistance, precision medicine, treatment response, actionable mutations

## Abstract

Global statistics have placed colorectal cancer (CRC) as the third most frequently diagnosed cancer and the fourth principal cause of cancer-related deaths worldwide. Improving survival for CRC is as important as early detection. Personalized medicine is important in maximizing an individual’s treatment success and minimizing the risk of adverse reactions. Approaches in achieving personalized therapy in CRC have included analyses of specific genes with its clinical implications. Tumour genotyping via next-generation sequencing has become a standard practice to guide clinicians into predicting tumor behaviour, disease prognosis, and treatment response. Nevertheless, better prognostic markers are necessary to further stratify patients for personalized treatment plans. The discovery of new markers remains indispensable in providing the most effective chemotherapy in order to improve the outcomes of treatment and survival in CRC patients. This review aims to compile and discuss newly discovered, less frequently mutated genes in CRC. We also discuss how these mutations are being used to assist therapeutic decisions and their potential prospective clinical utilities. In addition, we will summarize the importance of profiling the large genomic rearrangements, gene amplification, and large deletions and how these alterations may assist in determining the best treatment option for CRC patients.

## 1. Introduction

Colorectal cancer (CRC) is currently placed as the third most frequently diagnosed cancer and is ranked third in terms of mortality [1]. Its burden is anticipated to rise by 60%, which will result in more than 2.2 million new cases and 1.1 million cancer deaths by the year 2030 [2]. The rise in incidence is reported mainly from the low and middle-income countries, particularly in Asia [3,4]. The overall trend, however, has begun to stabilize or decrease in developed countries, including the United States, Canada, Australia, and north-western Europe, due to the implementation of screening and early detection programs [2,3]. The five-year survival rate is highly reliant on the disease stage upon diagnosis. Despite an excellent five-year survival rate for patients diagnosed with Stage I CRC (>90%), the survival rate reduced dramatically to merely 10% for patients diagnosed with Stage IV CRC [5]. Hence, early detection of the disease plays a significant role in getting better survival outcomes.

Treatment of CRC primarily consists of surgery, adjuvant chemotherapy, neoadjuvant radiotherapy, as well as targeted therapy. With the advancement in systemic treatments and newly developed biological drugs targeting either angiogenesis or epidermal growth factors (EGFRs), such as cetuximab and panitumumab, the overall survival has significantly increased, mainly in patients with metastatic CRC (mCRC) [6]. On top of that, immune checkpoint inhibitors have shown promising outcomes in a subset of patients with mCRC with microsatellite unstable hypermutated and mismatch repair deficient (dMMR) profiles [7]. Unfortunately, ineffective drug treatment and acquired resistance to therapy are believed to be a hindrance to better outcomes and contribute to low survival rates of CRC patients [5,8]. Multidrug resistance is one of the main reasons for treatment failure in more than 90% of patients with mCRC [9,10]. The monoclonal antibodies (mAbs) cetuximab and panitumumab are among the most common targeted therapies used in late-stage CRC. However, they are only effective in a small percentage of patients [11,12,13] due to either intrinsic or acquired resistance to this type of therapy. Unfortunately, even the patients that initially respond to EGFR antagonists usually acquired resistance over time [14,15,16]. Taken together, these findings necessitate a change in treatment and prediction approaches. A better understanding of the mechanism of inherent and acquired therapy resistance will be of important value for drug development, along with improved clinical outcomes.

## 2. Less Frequently Mutated Genes in CRC

**Figure 1:**
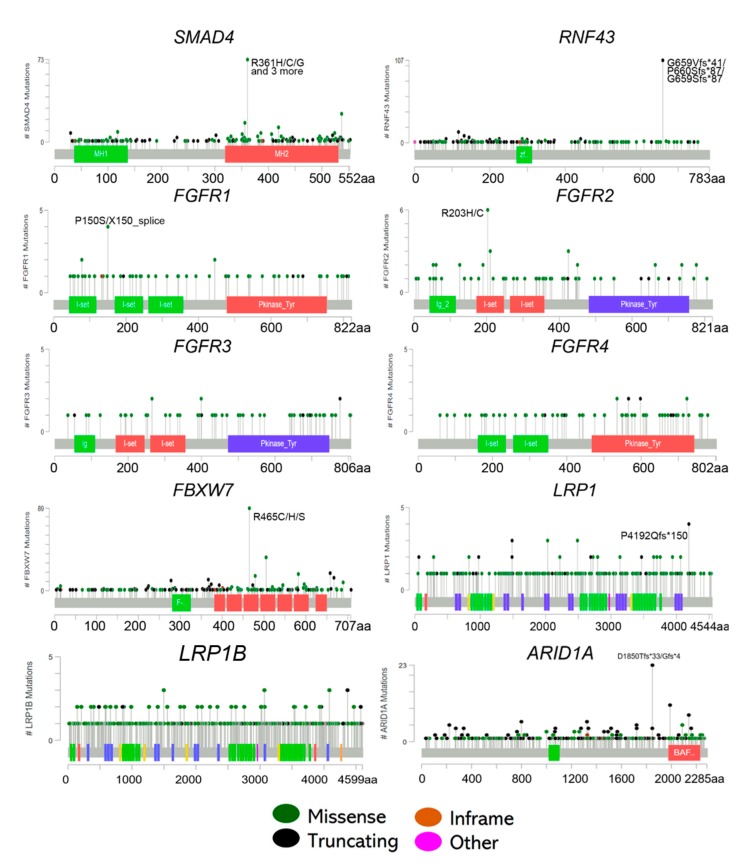
Lollipop plots of alterations in the less frequently mutated genes [34,52].

CRC is known to have a high inter-patient molecular heterogeneity. Given the advent of next-generation sequencing technology, common and rare somatic mutations in patients can be profiled specifically. Data on the molecular profiles of CRC are relatively increasing, and mutations are now well-characterized, but they are sometimes conflicting [17]. While there is massive data regarding *APC*, *KRAS*, *PIK3CA,* and *TP53* gene mutations, minimal attention has been given to less frequently mutated genes as they are mostly identified from several genomic approach research with a small number of CRC samples. Nevertheless, an increasing number of gene alterations have been discussed in terms of their roles in treatment stratification and how these alterations have been translated into drug development and promising positive predictive markers [18]. In Table 1, we summarize several research efforts to identify dependable new biomarkers to help clinicians make tailored treatment decisions in CRC. Some of these alterations are located in receptor tyrosine kinases (RTK) genes *(FGFR1, FGFR2, FGFR3,* and *FGFR4),* which have important implications for the selection of anti-cancer therapies [19,20]. Furthermore, several mutated genes were discovered to be involved in important pathways in CRC, including TGF-β family member signaling (i.e, *SMAD4)*) and the Wnt signaling pathway (*RNF43*). There are several reported cancer cases that did not display any mutations of known cancer driver genes [21]. However, these cancers exhibit a large set of genes mutated with intermediate (2%–20%) or low frequency (less than 2%) [21,22]. Collectively, this justifies the need for further exploration of how these alterations may play a role in tumorigenesis or treatment response. The distribution of alterations in the less frequently mutated genes is displayed in Figure 1.

In this review, we discuss newly discovered but less frequently mutated genes found in CRC. We will highlight how these mutations are presently used to assist treatment decisions and their prospects of being clinically valuable in the future. We will also review the importance of profiling the genomic rearrangements, mostly those involving gene amplification, in CRC and how these alterations may assist in determining the best treatment option for CRC patients.

## 3. *SMAD4* Mutations

The transforming growth factor-beta (TGF-β) signaling pathway is crucial in many important cellular processes such as differentiation, proliferation, apoptosis, and extracellular matrix production [53]. The activation of this pathway starts upon the binding of TGF-β ligand to cell surface receptor protein, known as TGF-β transmembrane protein kinase, and triggers the activation of a group of related SMAD proteins [54]. The SMAD protein is involved in transmitting signals from the cell surface to the nucleus. Alteration in this pathway is known to be associated with carcinogenesis and cancer progression of CRC. During the early stage of CRC, inactivation of TGF-β signaling is related to tumor suppression [55]. However, in the late stage of CRC, TGF-β causes tumor-promoting effects via its capability to cause epithelial–mesenchymal transition (EMT), which augments metastatic and invasion abilities [56]. On top of that, SMAD proteins may act as transcription factors as well as tumor suppressors by regulating the activity of genes involved in cell growth and proliferation [57]. Interaction between the TGF-β signaling pathway and several classical pathways such as MAPK (mitogen-activated protein kinase), PI3K/AKT (phosphatidylinositol-3 kinase/AKT) and WNT/β-catenin pathways have been discussed extensively [58]. TGF-β signaling was found to regulate the WNT/β-catenin pathway through the SMAD4 formation complex with β catenin and LEF [59]. The deletion of SMAD4 in a CRC cell line was proven to increase the mRNA levels of β-catenin and Wnt signaling, thus elucidating the interaction between TGF-β and the Wnt signaling pathway in CRC progression [60]. Wnt signaling in CRC can be activated through BMP signaling and it has been shown that 5FU chemosensitivity was influenced by BMP signaling, depending on SMAD4 and p53 mutation statuses. 

Somatic mutations in *SMAD4*, which is the most common compared to *SMAD2* and *SMAD3*, were known to be significantly involved in advanced or mCRC [61]. The loss of heterozygosity (LOH) on chromosome 18q has been proven to be associated with loss of *SMAD4* expression and has been reported in 95% of invasive and mCRCs with *SMAD4* somatic mutations. Conversely, adenoma and intramucosal carcinoma with wild type *SMAD4* gene harbor low frequencies of 18qLOH [62]. The loss of SMAD4 expression, due to this genetic aberration, has been predicted to be linked with poor prognosis in CRC. CRC patients with tumors expressing high SMAD4 levels have significantly better survival compared to patients with a low SMAD4 expression level [63].

High SMAD4 protein levels are also detected in microsatellite instable and hypermethylated CRCs and are associated with a better prognosis [64]. In a re-analysis of TCGA CRC cases, the high rate of SMAD4 and TGF-β pathway mutations is explained by microsatellite instability and hyper-mutation in a subset of tumors harboring defective DNA mismatch repair [26]. More recently, Yoo and colleagues have also proven this correlation whereby tumors overexpressing SMAD4 showed a significant association with sporadic microsatellite instability [65]. 

However, somatic mutations of *SMAD4* are less common as compared to the loss of heterozygosity and are identified in between 2% to 20% of CRCs [25]. Unique *SMAD4* mutations, as well as recurrent changes, with more than 60% of them being novel, were reported in 64 out of 744 sporadic CRC patients (8.6%) treated in hospitals across Australia. The mutations were predominantly pathogenic, with most missense alterations predicted to diminish protein stability or thwart the formation of the SMAD complex [61]. A low frequency of *SMAD4* mutation was also observed in patients of Iranian descent (2%, 1 out of 51). Upon validation, one novel heterozygous non-synonymous variant, R496C, c.1486C>T, was detected with a frequency of 0.08% (5 out of 63) and located at the MH2 region of the *SMAD4* gene. Nonetheless, due to the heterozygous nature of this validated variant, the potential impact on the oncogenic transformation was not assessed [66].

Even though somatic mutations in *SMAD4* are less common in CRC, the functionality of these mutations and how they affect treatment outcomes are currently being explored. Evidence from several studies pointed out that *SMAD4* is a predictive biomarker for 5-fluorouracil (5FU)-based chemotherapy in CRC patients [67,68,69]. The loss of function of *SMAD4* was found to be associated with resistance towards 5-FU based treatment through activation of the PIK3/Akt pathway. Interestingly, the PI3K inhibitor known as LY294002 was able to restore the chemosensitivity of CRC by inhibiting the PI3K/Akt/CDC2/survivin cascade [69]. The authors proposed *SMAD4* as a candidate biomarker for combined LY294002 and 5-FU-based chemotherapy regimens for patients with CRC.

On top of that, the response to anti-EGFR treatment in patients harboring *SMAD4* mutations is also being explored. In a study involving 734 CRC patients, 90 (12%) had *SMAD4* mutations, and the missense mutations at R361 and P356 in the MH2 domain were the most common *SMAD4* alterations, as verified by full-length sequencing. A subset of patients with mCRC with wild-type *KRAS*, *NRAS*, and *BRAF* who received anti-EGFR therapy were shown to have shorter progression-free survival (PFS) duration compared to patients with unmutated *SMAD4* [26]. Similarly, research by Mei et al. [25] showed that patients carrying *SMAD4* mutations had significantly shorter PFS compare to those carrying wild-type *SMAD4*. They also reported that none of the patients with *SMAD4* mutations were responsive to cetuximab at 12-week post-treatment. Taken together, the aberrance of *SMAD4* should be assessed when exploring targeted therapies for CRC patients.

## 4. *RNF43* Mutations

RING-type E3 ubiquitin transferase 43 (*RNF43*) is a type of ubiquitin ligase located in the transmembrane region [70]. *RNF43* acts as a tumor suppressor and negative regulator of Wnt/β catenin signaling, as well as non-canonical Wnt signaling [71]. Dysregulation of these pathways promotes tumorigenesis through several dysregulations of Wnt receptor ubiquitination. Frizzled protein and low-density lipoprotein receptor-related protein 5 or 6 (LRP5/6) are the main receptors of Wnt proteins, and binding of these proteins results in the formation of a specific complex of Frizzled and LRP5/6 receptors. Upon the binding of Wnt proteins to the receptors, the stabilized β-catenin proteins enter the nucleus, leading to the activation of the Wnt signaling pathway and target gene transcription, including the RNF43 gene. *RNF43* is involved in intermediating the ubiquitination, endocytosis, and, consequently, degradation of Wnt receptor complex components Frizzled. The ubiquitination leads to the elimination of Wnt receptors from the cell surface and, subsequently, inactivation of the Wnt signaling pathway [72]. In cancer, there are two distinct mechanisms to have continuously activated Wnt signaling. Firstly, through the loss of function of *RNF43* via mutations, which leads to decreased degradation of Frizzled with an augmented Wnt/β-catenin signaling pathway [72]. The second mechanism is by the silencing of TCF4 transcriptional activity. TCF4 is a partner of β-catenin and acts as a transcription factor of the Wnt signaling downstream gene. *RNF43* is found on the nucleus membrane and sequesters TCF4 to the nuclear membrane. Mutated RNF43, independent of its E3 ligase function, may lead to the release of TCF4, allowing it to act as a transcription factor [73].

In CRC, Wnt signaling is usually dysregulated via *APC* loss-of-function mutations, whereas *RNF43* was not significantly mutated in a previous sequencing study [23]. However, the *RNF43* gene is among the most frequently mutated gene in Wnt-dependent tumor types, such as CRC and endometrium cancer [28]. Through recent large scale genomic profiling of CRC via the whole-exome sequencing approach, *RNF43* was found to be significantly mutated in 488 non-hypermutated CRCs [74]. This is supported by in silico analysis of TCGA [23] data, whereby there are more than 18% of CRCs and endometrial carcinomas harbor somatic *RNF43* mutations [28]. The most commonly reported somatic mutations in CRC is a frameshift mutation at R117 (C6 repeat tract) in exon 3 and G659 (G7 repeat tract) in exon 9 [75]. These mutations have been identified in *BRAF* mutant/MSI sessile serrated adenoma and traditional serrated adenoma [76]. Moreover, Bond et al. [76] reported that *RNF43* is frequently mutated in 87% (47/54) *BRAF* mutant/MSI cancers. This is further supported by similar research done by Yan et al. [77], which identified more than half of the patients with *BRAF* V600E also acquiring aberrations in the Wnt pathway, including *RNF43* mutations. Truncating mutations of *RNF43* were also observed in colorectal adenocarcinoma, predominantly in microsatellite unstable cancers, and showed a mutual exclusivity pattern with inactivating APC mutations [28].

Quite recently, a loss-of-function study of *RNF43* in CRC cell lines (Colo205, SW620, HCT116, and HCT15) was conducted to explore the functional importance of *RNF43* mutations and the relationship with pathological characteristics as well as prognoses [29]. However, this was limited to the hotspot mutation p. G659fs and p. R117fs. To date, the influence of the mutations against standard therapy, such as 5-FU or oxaliplatin, has not been investigated. As mentioned previously, *RNF43* mutations were usually found to co-occur with a *BRAF* V600E mutation. Collectively, this data clearly implies that genetic alterations in the upstream Wnt pathway regulators lead to pathway activation and plays a major role in *BRAF* V600E colorectal carcinogenesis. Therefore, drug combinations that target both the MAPK and Wnt pathways could be an effective treatment approach in *BRAF*-mutated CRC patients. For instance, co-targeting ligand-dependent Wnt pathway activation in combination with *BRAF* or co-inhibition of *BRAF* and *EGFR* represents an intriguing potential therapeutic strategy [75]. Nonetheless, to maximize the benefit of targeted cancer therapeutics, it is critical to identify those patients who are more likely to respond to the therapy.

Somatic *RNF43* alterations have also been linked to increased sensitivity towards compounds targeting the Wnt pathway, such as a specific small molecule of porcupine inhibitor, LGK974, in preclinical models [78]. In one study [79], this drug reduced the invasion and increased apoptosis in two of CRC cell lines, namely, SW742 and SW480. The study also illustrates the deregulation Wnt pathway-related genes as well as increased expression in several genes involved in MAPK and apoptosis pathway in LGK974 treated cells as compared to oxaliplatin. According to a study by Jiang and colleagues [30], the pancreatic ductal adenocarcinoma (PDAC) cell line with inactivating *RNF43* mutation is sensitive towards LGK974 treatment. However, not all PDAC cell lines are sensitive to LGK974. PDAC cell lines carrying homozygous mutations of *RNF43* were provn to confer resistance against LGK974, suggesting that there are alternative mechanisms involved [30]. Thus, the response of specific somatic mutations of *RNF43* against this inhibitor, particularly in CRC, remained to be explored and justify the need for detailed functional studies.

## 5. FGFR Mutations

Most of the tyrosine kinase receptors (TKR) share intracellular signaling pathways; hence, cancer cells have a propensity to resist the inhibition of one tyrosine kinase receptor by activating another. Therefore, in targeting TKRs, a multi-targeted tyrosine kinase inhibitor (TKI) that targets different TKRs at once is an interesting future prospect [80]. The fibroblast growth factor receptor (FGFR) family (*FGFR1-4*) comprises of TKRs implicated in several fundamental biological roles such as angiogenesis, embryogenesis, wound repair, and tissue homeostasis [81]. In a study of almost 5000 various cancers by next-generation sequencing, *FGFR* alterations were found in 7.1% of the cancers, with the majority being gene amplification (mostly in *FGFR1*), followed by mutations and rearrangements. Almost all types of cancers included in the study showed some patients with *FGFR* alterations, and the urothelial cancers were most affected. Meanwhile, only 4% of CRC patients in the study harbored *FGFR* alterations [35]. Taken together, these data suggest that *FGFR* might be an ideal candidate for therapeutic targeting across multiple cancer types.

*FGFR* alterations demonstrated the oncogenic potential through activating somatic mutations resulting in cell growth and conferring resistance to cancer therapy [80,82]. These alterations may lead to either constitutive activation of the receptor or decreased sensitivity to ligand binding. Point mutations in the kinase insert (KI) domain of *FGFR2* may lead to a conformational switch that enhances the kinase activation. Among them were P583L in CRC, G584V/W and I591M in lung cancer, M585V in cervical cancer, and S588C in breast cancer, which are all believed to be involved in oncogenesis via the deregulation of the pathway through aberrant FGFRs [83]. On top of that, fusion proteins that resulted from translocation events can cause isoform switching and reduced specificity towards fibroblast growth factors (FGFs) [84]. *FGFR2* amplification in CRC was identified in a CRC cell line, NCI-H716, as reported by Mathur et al. [37]. The same study revealed that FGFR-selective small molecules inhibitors were able to inhibit the cell viability in vitro as well as in a xenograft model. Nevertheless, *FGFR2* amplification was not observed in a subset of primary CRC tissues despite its overexpression. The findings indicate that *FGFR2* amplification is not prevalent in common types of CRC or lymph node and liver metastases. Yet, it remains plausible that distinct subsets, for instance, those with ascites or tumors with endocrine differentiation, which is the primary source of the NCI-H716 cell line, may have some frequency of amplification [37].

*FGFR* were implicated in resistance to conventional therapies in several cancers such as breast cancers [85], non-small cell lung carcinomas (NSCLC) [86], and melanomas [87]. To overcome this, a collaborative effort to develop FGF/FGFR inhibitors as anticancer treatments is underway, and some have entered the clinical phase [88]. In a panel of CRC cell lines with intrinsic resistance to oxaliplatin or 5FU, a synergistic interaction between silencing *FGFR4* and these therapies was demonstrated to reduce the cell growth and survival. This finding suggests the potential value of *FGFR4* as a targetable regulator in chemo-resistance in CRC [89]. As previously mentioned, the alterations of the *FGFR* gene are relatively rare in CRCs as compared to other cancers. Additionally, due to the wide spectrum of *FGFRs* alterations from mutations, amplifications to rearrangements, categorizing patients that are more likely to be responsive to FGFR inhibitors might be challenging. This highlights the need for further development of optimal molecular diagnosis screening for *FGFR* alterations, inclusive of next-generation sequencing, chromogenic in situ hybridization (CISH), fluorescent in situ hybridization (FISH), or quantitative real-time PCR.

## 6. *FBXW7* Mutations

F-box WD repeat domain-containing-7 (*FBXW7*) encodes for the F-box protein with seven tandem WD40 and is located at chromosome 4q31.3. It is one of the vital substrate-recognition subunits of ubiquitin ligase called the Skp1-Cdc53/Cullin-F-box-protein complex (SCF/β-TCP) [90,91]. The Catalogue of Somatic Mutations in Cancer (COSMIC) database identified that *FBXW7* has the highest frequency of mutation in both F-box and WD repeat domain-containing family members and SCF ubiquitin ligase complexes, with a mutation percentage of 2.54 % [92]. The FBXW7 protein is considered as a potent tumor suppressor [92] since the majority of its target substrates acts as potential growth promoters (proto-oncogenes), including c-Myc, c-JUN, cyclin E, Notch, and KLF5 [93,94,95]. Therefore, any deletion, mutations, or hypermethylation in the human *FBXW7* gene could lower or inactivate *FBXW7*, resulting in the build-up of oncogenic substrates, which could lead to the formation and progression of various cancers, including CRC [96,97].

Until today, *FBXW7* has been constantly recognized as one of the less commonly mutated genes in CRC, accounting for approximately 6% to 15% of all cases [20,39,41,98]. The mutational range of *FBXW7* in CRC is somewhat peculiar, with over 70% of missense single nucleotide variations affecting amino acids in the substrate-binding sites, and the most common mutational hotspots are at the two important arginine residues at the position 465 and 479 (Arg^465^ and Arg^479^) [39,99]. The remainder is mostly nonsense alterations, which lead to premature termination of *FBXW7* translation, while the loss of an entire allele is a rare occurrence [93]. In a study conducted in 2015, profiling of CRC displayed a missense mutation of *FBXW7* in chromosome 4 (position: 153247289) with a change in the amino acid sequence R425C [100]. Later in 2017 [44], Korphaisarn et al. identified *FBXW7* mutations in 43 out of 571 CRC patients. Among them, 37 patients had missense alterations (R465C, R465H, and R505C), four had nonsense alterations, and the remaining two harbored frameshift alterations. Missense mutations could also occur at S582L, affecting Ser^582^. Based on the results, not only were these missense mutations in *FBXW7* associated with poor overall survival and prognosis but also demonstrated resistance to oxaliplatin, especially in the metastatic patients [44]. Additionally, there was no difference in the mutation frequency of *FBXW7* between primary and metastatic patients. Taken together, these data suggested that missense alterations in a single allele of *FBXW7* impaired its activity, but there is still insufficient data to validate any pathological clinical or demographic features as the representative of the patients with *FBXW7* mutations [39,92]. In short, although *FBXW7* mutations showed promise as the negative prognostic marker in CRC, additional investigations are necessary to discover downstream pathways causing this worse prognosis as well as its value as a predictive biomarker for drug response.

## 7. *LRP1* Mutations

The low-density lipoprotein receptor (LDLR)-related protein 1 (*LRP1*) is a family member of the low-density lipoprotein receptor (LDLR), which serves as a multifunctional endocytic receptor in two major cell processes; endocytic and signalling activities [101]. This large and ubiquitously expressed transmembrane receptor recognizes numerous ligands, including growth factors. Thus, *LRP1* is known to regulate various cell functions, such as lipoprotein metabolism and cell motility [102,103]. In cancer, *LRP1* was suggested to play a dual role in cell invasion and migration, depending on the specific cell type and their microenvironment [104]. *LRP1′s* role might vary from one tumor type to another. *LRP1* expression levels are often deregulated and reported to be related with advanced tumor stage and poor prognoses in several cancers, such as CRC [46], lung adenocarcinoma [105], melanoma [47], and hepatocellular carcinoma [106]. On the other hand, high *LRP1* expression was reported in the advanced tumor stage of astrocytoma [107], endometrial [108], and breast cancer [109], further suggesting conflicting roles of this gene, which warrant future research.

Among the reported *LRP1* mutations are the polymorphic alleles of C766T in exon 3 of the gene that was reported several decades ago in astrocytoma [107]. Nevertheless, there is no significant difference in terms of the frequency of C766T as compared to the controls [107]. Moreover, the same study also reported that *LRP1* gene amplification in occurrence with EGFR amplification was observed in high-grade astrocytomas (Grade IV), compared to normal brain tissues. These data might suggest that amplification of the gene may be partly involved in the high expression of *LRP1* mRNA. Later, the same mutation of C766T was also identified in breast cancer patients, whereby the frequency of T-allele was high in breast cancer patients compared to the control population, suggesting its link to an increased in breast cancer risk [110]. Analysis of the TCGA CRC dataset showed that *LRP1* gene mutation is uncommon, accounted for only 6% of the cases [23,46]. Low mRNA expression of the gene in the *LRP1* mutated group compared to the wild-type group was observed [46]. The same study also revealed that the decrease of mRNA expression was not due to the methylation of the gene’s promoter. A low level of mRNA expression was found to be correlated with poor prognosis, mainly among Stage IV CRC patients [46]. Hence, although rare, the mutations may partially justify the reduction in *LRP1* mRNA expression and poor clinical outcomes in some CRC patients.

The roles of *LRP1* in cancer cells have been widely investigated in some cancer cell lines such as glioblastoma [111] and thyroid cancer cell line [112]. In glioblastoma cells, *LRP1* was reported to regulate the expression of *MMP-2* and *MMP-9,* which are responsible for promoting the migration and invasion of the cells. In addition, the level of phosphorylated ERK was decreased in *LRP1*-deficient cells, whereas other signaling pathways remained unchanged, suggesting that *LRP1* possibly regulates the expression of *MMP-2* and *MMP-9* via an ERK-dependent signaling pathway, resulting in cell migration and invasion [111]. The role of *LRP1* in cancer cell invasion and migration is, however, controversial as some of the findings demonstrated that low expression of *LRP1* can also promote tumor cell progression [104]. These findings suggest that profiling of either mutation or expression profile of *LRP1* is crucial in determining the impact on specific cell types. In CRC, the mechanism on how the mutations regulate *LRP1* expression and the impact of *LRP1* expression remain unknown so far. Taken together, any mutations in *LRP1* might probably lead to deregulation of the mRNA expression level and could potentially serve as a biomarker, which warrants further research.

The low-density lipoprotein receptor-related protein 1B (*LRP1B*) is closely related to *LRP1*. In CRC, *LRP1B* down-regulation enhanced CRC cells growth and migration. Additionally, knocking down of *LRP1B* increased the expressions of several target genes downstream of beta-catenin/TCF signalling which are Cyclin D1, N-cadherin, and Snail, thus promoting metastasis in CRC [113]. Therefore, restoring the function of *LRP1B* would be a promising therapeutic approach for CRC.

With regard to the mutational landscape, *LRP1B* alteration frequency in CRC is strikingly different from *LRP1*. Single-cell DNA sequencing proved the presence of *LRP1B* mutations in mCRC [114]. In 2018, Cybulska et al. [115] revealed that *LRP1B* mutations account for 46% out of the 2832 single-nucleotide variants and short indels included in the study. In the same year, Wolf et al. [116] identified 25% of *LRP1B* mutations among the 148 CRCs screened. However, to date, there is no scientific evidence on the influence of the mutations of *LRP1B* in CRC towards its diagnosis or prognosis. Since knockdown of *LRP1B* leads to promoted growth, migration, and metastasis in CRC, any mutations resulting in the functional loss of *LRP1B* could act as a CRC prognostic marker, but additional functional studies are needed for validation.

## 8. *ARID1A* Mutations

AT-rich interactive domain 1A, known as *ARID1A*, is a component of the switching defective/sucrose non-fermenting (SWI/SNF) chromatin remodeling complex, which involves gene expression regulation [117]. *ARID1A* mutations and loss of its expression were observed in ovarian clear cell cancer [118], endometrioid cancer [119], breast cancer [120], Burkitt lymphoma [121], and lung cancer [122]. However, the investigation of this gene in the CRC is limited, and the mechanism by which the inactivation of the gene involved in tumorigenesis is not clearly understood [49].

A group of researchers has utilized the patients’ data from the Cancer Genome Atlas (TCGA), Nurses’ Health Study and Health Professionals’ Follow-Up Study (NHS/HPFS), AACR Project GENIE, and MD Anderson Cancer Center databases to characterize the *ARID1A* mutations in CRC. From a total of 3127 patients, 196 (6.2%) had at least one mutation in *ARID1A*. In the same dataset, 249 mutations across the gene were identified; most of the mutations were frameshift or nonsense mutations [48], which may lead to protein truncation and loss of ARID1A protein expression. The prevalence of the *ARID1A* mutation and the loss of protein expression was reported by approximately 12%–13% through a meta-analysis approach. Remarkably, the loss of ARID1A protein expression in CRC patients was significantly associated with poorly differentiated grade and advanced tumor depth [123], suggesting the loss of ARID1A protein expression as a predictive marker for poor prognosis CRC. However, some conflicting data exist, according to which the loss of ARID1A by immunohistochemistry was higher in primary CRCs with a frequency of 25.8% [49]. A higher prevalence of *ARID1A* mutation was observed in 18 out of 46 (39%) microsatellite instable (MSI) CRC, with almost half of them harboring the hotspot mutation c.5548delG7, indicating this mutation may play a role in MSI CRC [124].

It was reported that the *ARID1A* homolog, which is *ARID1B,* is required for the survival of *ARID1A*-mutant cancer cell lines. The silencing of the *ARID1B* gene in a *ARID1A*-mutated ovarian clear cell carcinoma line destabilized SWI/SNF and impaired the proliferation of the cells [125]. This indicates that the presence of *ARID1B* is necessary for stabilizing the SWI/SNF complex in *ARID1A*-mutant cancer cells. Additionally, the low *ARID1B* expression level in *ARID1A*-mutated patients was associated with shorter progression-free survival, suggesting that a low *ARID1B* level could be a marker of poor prognosis in OCCC with *ARID1A* mutations [126]. Recently, the depletion of *ARID1B* has also been proved to increase radiosensitivity in an *ARID1A* mutant CRC cell line, providing a new perspective for targeting *ARID1B* in combination with radiotherapy to enhance outcomes of patients with *ARID1A*-mutant CRC patients [51].

The involvement of *ARID1A* in regulating chemoresistance in CRC has been explored by overexpressing and silencing of this gene. Reduced ARID1A expression promotes cell proliferation and suppresses 5-FU-induced apoptosis in an SW620 CRC cell line. Meanwhile, the depletion of ARID1A in SW480 cells enhanced the proliferation and inhibited apoptosis upon 5-FU treatment [50]. Nevertheless, the depletion of ARID1A was performed by a siRNA approach, not by introducing mutations that may cause loss of *ARID1A* mutation. In another study, a knockout *ARID1A* CRC model was generated using a CRISPR/Cas9-mediated gene editing approach in the CRC cell line harboring KRAS mutation [127]. Without ARID1A, the proliferation of these cell lines is seriously impaired, indicating that ARID1A plays an essential role. On top of that, loss of ARID1A may lead to disruption of KRAS/AP1-dependent enhancer activity, affecting the expression of target gene MEK/ERK pathway [127]. Collectively, the relationship between either *ARID1B* or *KRAS* and the mutation *ARID1A* presents a unique potential for the development of novel combination therapeutic approaches in precision medicine.

## 9. Co-occurrence of the Less Frequently Mutated Genes

Cancers are polygenic diseases partly caused by various genomic changes that result in loss of cell division regulation. Such changes contribute to one another in patterns of mutual exclusivity or co-occurrence that influence prognosis and response to treatment. Many cases of co-occurring genomic changes have been reported, indicating that certain alterations in the related pathways lead to complementary, rather than duplicate, effects [128]. Using the cBioportal tool [34,52], a combination of the genes from Table 1 revealed that most of those less frequently mutated genes are concurrently altered in CRC. Table 2 illustrates the significant co-occurrence feature of these genes in 3806 CRC patients from 10 TCGA studies (http://bit.ly/2TJwIce). 

## 10. Other Genomic Alterations: Large Genomic Rearrangement and Deletions

Extensive research has focused on interrogating somatic point mutations in relation to their clinical impact. However, there are several cancers that are driven by structural variants (SVs) or copy number alterations (CNAs) [129]. In Lynch syndrome, large genomic rearrangements of the mismatch repair (MMR) genes have been reported, with a variable frequency, depending on the population studied, from 5% to 20% [130], and with *MLH1* and *MSH2* being the most affected genes [131]. A novel large deletion in the *MSH2* gene that resulted from Alu-mediated arrangement has been reported in one of the Southern Italian patients (1.6% frequency) with an inherited predisposition to CRC [132]. Even though it was a rare incident, identification of the alterations may rule out the negative point mutation in MMR genes of the Lynch syndrome patients, which is important to family members.

Most of the CNAs identified in CRC were either amplification of oncogenes or deletion of tumor suppressor genes, such as *MYC*. The prevalence of *MYC* amplification of 8% to 25% was observed in several studies [133,134]. This alteration was proven as an independent factor to be associate with poor prognosis in CRC patients. However, other groups proved otherwise. A meta-analysis study done in 2018 [135] and in a study of 334 Korean CRC patients [136] conclude that the cumulative amplification status of *MYC* had no correlation with the outcome of patients. Collectively, these findings indicate the uncertain role of *MYC* amplification in predicting the patients’ outcomes, which warrant further investigation.

Due to gene amplification, overexpression of *MYC* may result in the activation of several downstream genes, leading to a promotion of the cell cycle with DNA synthesis and an increase in chromosomal aberration. These pathways can ultimately cause genomic instability and chemoresistance [137]. Several promising *MYC* inhibition strategies in CRC have been explored. *MYC* inhibition and resistance to chemotherapy were investigated through the development of a novel 3D organoid culture model from the CRC patient. Hedgehog signals are involved in regulating the nuclear translocation of GLI-1, which triggers the transcription of target genes, including *MYC*. Combination therapy with hedgehog-inhibiting agents such as AY9944, GANT61 and 5-FU, irinotecan, or oxaliplatin, decreased cell viability of CRC organoids compared to single treatment [138]. Taken together, the identification of selective *MYC* inhibitors is necessary in order to develop more effective and less toxic therapeutic agents that can be used either alone or in combination with conventional therapy.

## 11. Future Recommendations and Conclusions

Although the frequency of mutation in each gene discussed in this review was comparatively low, based on the evidence listed in Table 1, all of them are hypothetically pertinent for the prognostic assessment and identification of patients suitable for targeted therapies. Furthermore, based on TCGA findings, 40% of TCGA patients harbor alterations in at least one of these genes [52], highlighting its cumulative effect. It will be interesting to examine how the co-occurrence of alterations in the less frequently altered genes will influence overall survival or disease-free survival, as well as the response to chemotherapy.

CRC is a heterogeneous disease with many diverse sets of alterations in tumor suppressor genes and oncogenes. With the advancement in next-generation sequencing, whole-genome sequencing enables the profiling of the whole spectrum of genetic changes, including copy number alterations and structural variants, hence refining the discovery of reliable biomarkers of chemo-responsiveness or chemoresistance against targeted treatment in CRC. Finally, in our opinion, a comprehensive molecular characterization, including the less frequently mutated genes, in combination with a better understanding of the genes’ function, are necessary before this can be translated into clinical practice to improve the management of CRC patients.

## Figures and Tables

**Table 1:** Less frequently mutated genes with treatment implications and their roles in either in vitro or in vivo.

Altered Gene	Prevalence in CRC	Actionable and/or Predictive Value	Highest Level of Evidence	In vitro or In vivo Investigation in CRC and/or Other Cancers
*SMAD4*	2%–20% [23,24]	Resistance to anti-EGFR monoclonal antibodies, cetuximab as a single agent or in combination with standard chemotherapeutic agents [25].	Retrospective Cohorts	*SMAD4* deficiency induces 5 fluorouracil (5FU) chemoresistance in CT26 and SW620 cells via the activation of PI3K/Akt/CDC2/survivin pathway. The PI3K inhibitor, LY294002, able to trigger 5FU chemosensitivity via cell cycle arrest by hindering the PI3K/Akt/CDC2/survivin cascade in the *SMAD4*-deficient cells [27].
		Unresponsive to anti-epidermal growth receptor therapy and significantly shorter-progression-free survival durations [26].	Retrospective Cohorts	
*RNF43*	6%–18% [28,29]	Sensitive to LGK974 for pancreatic cell line with *RNF43* loss of function mutation [30].	Case Study	*RNF43* knockdown enhances the tumorigenic potential of CRC cell lines in vitro and *in vivo*. Larger tumors were observed in the *RNF43* knockout mouse model [32].
		Phase I evaluation of LGK974 in melanoma, breast cancer (lobular or triple-negative) and pancreatic cancer [31].	Phase I Clinical Trial	
FGFRs	None was reported in one CRC study [33]; however, TCGA studies reported 1.7%–5% of CRC patients harbored alteration in FGFR genes [34]	Sensitive to *FGFR* Tyrosine Kinase Inhibitor (TKIs), AZD4547, as reported by Phase I and II clinical trials in gastric cancers [36].	Phase II Clinical Trial	*FGFR2* amplification and overexpression were implicated in survival and proliferation of CRC cell line NCI-H716 and sensitive to *FGFR* inhibitors [37].
	In other cancers: *FGFR1*: 3.5% *FGFR2*: 1.5% *FGFR3*: 2.0% *FGFR4*: 0.5% [35]	*FGFR* tyrosine-kinase inhibitors (TKIs), AZD4547, demonstrated growth inhibition in the colorectal cell line with *FGFR2* amplification [37].	Preclinical	
*FBXW7*	6%–20% [20,38,39,40,41]	Sensitive to mTOR inhibitors rapamycin in breast cancer cell line with the loss of *FBXW7* and deletion or mutation of *PTEN* [42].	Preclinical	Mutated CRC cell lines are less sensitive to regorafenib and sorafenib [45].
		Better clinical outcome in T-cell acute lymphoblastic leukaemia (T-ALL) patients [43].	Clinical	
		mCRC patients harboring *FBXW7* missense mutations had significantly worse overall survival than those with wild-type *FBXW7* [44].	Retrospective Cohorts	
LRP1	6% [23,46]	mCRC patients with mutations and low expression of LRP1 had poor clinical outcomes even though after treatment with bevacizumab [46].	Retrospective Cohorts	LRP1 together with its ligands, tissue plasminogen activator (tPA), regulate melanoma growth and lung metastasis in vivo [47].
*ARID1A*	6.2%–10.9% [34,48]	*ARID1A* protein loss, due to mutations, is associated with the late TNM stage, distant metastasis, and poor pathologic differentiation in CRC patients [49]	Retrospective Cohorts	*ARID1A* overexpression in SW620 cell line inhibits proliferation and facilitated 5-FU-induced apoptosis. *ARID1A* knockdown in SW480 cell line promotes proliferation and inhibited 5-FU-induced apoptosis [50].
		Stage IV patients with *ARID1A* protein loss in primary tumors had longer survival compared to those with *ARID1A* positive tumors [49]		CRC cell lines with mutated *ARID1A* are are selectively sensitized to ionizing radiation after knockdown of its other subunit, *ARID1B* [51].

**Table 2:** Significant co-occurrence of the less frequently mutated genes.

Gene A	Gene B	Log_2_ Odds Ratio	q-Value	Tendency
*ARID1A*	*FGFR3*	>3	<0.001	Co-occurrence
*RNF43*	*FGFR2*	>3	<0.001	Co-occurrence
*RNF43*	*FGFR3*	>3	<0.001	Co-occurrence
*LRP1*	*FGFR2*	>3	<0.001	Co-occurrence
*ARID1A*	*FGFR2*	2.99	<0.001	Co-occurrence
*FGFR2*	*FGFR1*	2.784	<0.001	Co-occurrence
*RNF43*	*LRP1*	2.618	<0.001	Co-occurrence
*FGFR3*	*FGFR4*	2.6	<0.001	Co-occurrence
*FGFR2*	*FGFR4*	2.532	<0.001	Co-occurrence
*ARID1A*	*RNF43*	2.503	<0.001	Co-occurrence
*FGFR2*	*FGFR3*	2.413	0.001	Co-occurrence
*LRP1*	*FGFR3*	2.411	<0.001	Co-occurrence
*RNF43*	*FGFR4*	2.344	<0.001	Co-occurrence
*LRP1B*	*FGFR3*	2.339	<0.001	Co-occurrence
*ARID1A*	*LRP1*	2.202	<0.001	Co-occurrence
*FGFR1*	*FGFR3*	2.02	<0.001	Co-occurrence
*FGFR1*	*FGFR4*	1.974	0.001	Co-occurrence
*FBXW7*	*FGFR3*	1.926	<0.001	Co-occurrence
*FBXW7*	*FGFR2*	1.913	<0.001	Co-occurrence
*LRP1*	*FGFR4*	1.737	0.004	Co-occurrence
*LRP1*	*LRP1B*	1.651	<0.001	Co-occurrence
*FBXW7*	*LRP1*	1.568	<0.001	Co-occurrence
*RNF43*	*FGFR1*	1.459	<0.001	Co-occurrence
*ARID1A*	*LRP1B*	1.447	<0.001	Co-occurrence
*LRP1B*	*FGFR2*	1.41	0.005	Co-occurrence
*FBXW7*	*FGFR4*	1.318	0.002	Co-occurrence
*LRP1*	*FGFR1*	1.316	0.005	Co-occurrence
*LRP1B*	*FGFR4*	1.247	0.016	Co-occurrence
*ARID1A*	*FBXW7*	1.216	<0.001	Co-occurrence
*RNF43*	*LRP1B*	1.186	<0.001	Co-occurrence
*LRP1B*	*FGFR1*	1.121	0.004	Co-occurrence
*FBXW7*	*RNF43*	1.111	<0.001	Co-occurrence
*ARID1A*	*FGFR4*	1.031	0.032	Co-occurrence
*ARID1A*	*FGFR1*	0.988	0.004	Co-occurrence
*FGFR4*	*SMAD4*	0.969	0.018	Co-occurrence
*FBXW7*	*LRP1B*	0.905	<0.001	Co-occurrence
*FGFR3*	*SMAD4*	0.824	0.036	Co-occurrence
*FGFR1*	*SMAD4*	0.726	0.01	Co-occurrence
*FBXW7*	*FGFR1*	0.691	0.016	Co-occurrence
*LRP1B*	*SMAD4*	0.637	0.006	Co-occurrence
